# Supplementary material for: Human xenobiotic metabolism proteins have full-length and split homologs in the gut microbiome
Source: bioRxiv. 2024 Nov 8:2024.11.06.622278. Preprint. [Version 1] doi: 10.1101/2024.11.06.622278 (PMC11580864; doi:10.1101/2024.11.06.622278)
Supplement: 1 [file NIHPP2024.11.06.622278v1-supplement-1.pdf]

bioRxiv preprint doi: https://doi.org/10.1101/2024.11.06.622278; this version posted November 8, 2024. The copyright holder for this preprint (which was not certified by peer review) is the author/funder, who has granted bioRxiv a license to display the preprint in perpetuity. It is made available under aCC-BY 4.0 International license.

Supplementary Tables and Figures

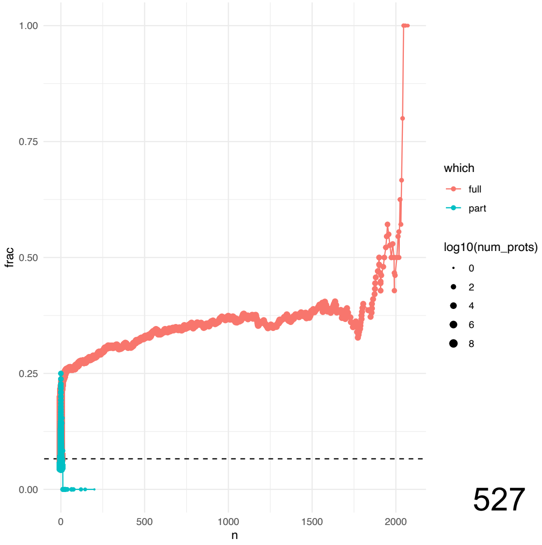

Supplementary Figure 1: Trend in mitochondrial localization for full-length (orange) and split (teal) homologs, as a function of their distribution across gut species. Each dot represents all microbial homologs present in at least a certain number of gut microbial species (x-axis). The size of the dot corresponds to the total number of such microbial homologs. The y-axis shows what fraction of the human homologs are annotated as localizing to the mitochondrion. The overall rate for all proteins is shown by the dashed line.

Supplementary Table 1: GO term enrichment (biological process) for human proteins with more full-length than split homologs. Terms with adjusted p-values below 0.05 are shown.

Supplementary Table 2: GO term enrichment (biological process) for human proteins with more split than full-length homologs. Terms with adjusted p-values below 0.05 are shown.

Supplementary Table 3: Drugs metabolized by human proteins with mostly full-length homologs in the gut microbiome.
